# Extended Long‐Term Effects of Cognitive and Aerobic Training on Cognitive Function in Patients With Stress‐Related Exhaustion Disorder: A 4.5‐Year Follow‐Up of a Randomized Controlled Trial

**DOI:** 10.1002/smi.70218

**Published:** 2026-09-04

**Authors:** Ingela Aronsson, Anna Stigsdotter Neely, Carl‐Johan Boraxbekk, Therese Eskilsson, Hanna M. Gavelin

**Affiliations:** ^1^ Department of Psychology Umeå University Umeå Sweden; ^2^ Department of Health Education and Technology Luleå University of Technology Luleå Sweden; ^3^ Department of Social and Psychological Studies Karlstad University Karlstad Sweden; ^4^ Institute for Clinical Medicine Faculty of Medical and Health Sciences University of Copenhagen Copenhagen Denmark; ^5^ Institute of Sports Medicine Copenhagen (ISMC) and Department of Neurology Copenhagen University Hospital Bispebjerg Copenhagen Denmark; ^6^ Department of Public Health and Clinical Medicine Section for Sustainable Health Umeå University Umeå Sweden; ^7^ Department of Community Medicine and Rehabilitation Physiotherapy Umeå University Umeå Sweden

**Keywords:** aerobic function, clinical burnout, cognitive function, exhaustion disorder, long‐term effects, stress‐related ill health

## Abstract

Stress‐related conditions like clinical burnout and exhaustion disorder (ED) are associated with enduring cognitive problems. We have previously demonstrated that the addition of computerised cognitive training (CCT) and aerobic training (AT) to a multimodal rehabilitation programme (MMR) yielded greater improvements in cognitive function compared to MMR alone in patients with ED. These effects were maintained at the 1‐year follow‐up for CCT, but not for AT. Building on these findings, the present study examined the extended long‐term effects of CCT and AT on cognitive function, psychological health, and work ability, 4.5 years after the interventions. Participants were recruited from a stress‐rehabilitation clinic and 56 of the initial 132 participants returned for the 4.5‐year follow‐up. Assessments were conducted before (T1), immediately after (T2), 1‐year after (T3) and 4.5‐years after (T4) the interventions. Mixed model analyses assessed changes in the intervention groups relative to the control group from T1 to T4, with follow‐up comparisons examining within‐group stability of outcomes between T3 to T4. The primary outcome was cognitive performance on a global cognitive score. Secondary outcomes included domain‐specific cognitive functioning, self‐reported cognitive function, psychological health (burnout, depression, anxiety, and fatigue), and work ability. The analysis revealed sustained long‐term effects on the global cognitive score, a trained updating task and episodic memory in the CCT group, with stable performance between one‐ and 4.5‐year follow‐up. The addition of AT did not yield any extended long‐term effects on cognitive performance. Both groups showed extended long‐term improvements in self‐reported memory problems, although findings were mixed. Extended long‐term improvements were observed on burnout for both groups, with additional effects on anxiety and work ability in the AT group. Notably, these effects were not present at the 1‐year follow‐up and are more plausibly explained by selective attrition rather than a delayed intervention effect. In conclusion, the result indicates that cognitive interventions such as CCT can have lasting positive effects on cognitive function in patients with ED, whereas the long‐term psychological effects should be interpreted with caution.

**Trial Registration:**

ClinicalTrials.gov: NCT0073772

## 1 Introduction

Stress‐related ill health is a significant problem in many western countries, leading to societal costs through increased healthcare consumption and reduced workforce productivity (Hassard et al. 2018). Various classifications and diagnoses, including burnout, clinical burnout, neurasthenia, and exhaustion disorder, are used worldwide to describe stress‐related ill health. The overlap between these conditions is significant (Grossi et al. 2015; Maslach et al. 2001; van Dam 2021) and the diagnosis exhaustion disorder (ED) has been described as the clinical manifestation of burnout (Grossi et al. 2015). ED was introduced in the Swedish version of ICD‐10 in 2005 (see Appendix 1 for diagnostic criteria) and manifests through both physical, emotional, cognitive, and behavioural symptoms (Grossi et al. 2015; Lindsäter et al. 2023).

Cognitive dysfunction is prevalent in psychopathology as well as in stress‐related disorders. Prior research has shown that people with stress‐related disorders often express concerns of cognitive difficulties in everyday life and perform worse than healthy controls in various cognitive tasks (Deligkaris et al. 2014; Lindsäter et al. 2023; Nelson et al. 2021). A meta‐analysis by Gavelin and colleges showed that stress‐related illness was related to suboptimal performance across multiple cognitive domains such as episodic memory, working memory, executive functioning, attention and processing speed; where deficits were more salient under high cognitive load conditions (Gavelin et al. 2021). Although cognitive impairments tend to improve with time (Beck et al. 2013; Österberg et al. 2012; Österberg et al. 2014), both self‐reported and performance‐based measures suggest that former patients continue to experience persistent cognitive problems long‐term, lasting for 1.5 up to 7 years (Dalgaard et al. 2020; Eskildsen et al. 2016; Glise et al. 2020; Jonsdottir et al. 2017; Oosterholt et al. 2016). Despite this, there are surprisingly few studies on how to best support cognitive functioning across time in stress‐related disorders.

Cognitive function can be supported in different ways (Wilson 2008), with two common non‐pharmacological approaches being computerised cognitive training (CCT) and aerobic training (AT). CCT involves structured activities aimed at improving cognitive functions, either by strengthening underlying abilities (process training) or by teaching effective strategies for task performance (Bahar‐Fuchs et al. 2013; Teixeira‐Santos et al. 2019). In contrast, AT consists of structured exercise programs designed to improve cardiovascular fitness, health and cognitive function, including activities such as brisk walking, running, cycling, and swimming (Boa Sorte Silva et al. 2024), typically of moderate intensity (Erickson et al. 2019). Effects of CCT have been studied in normal and pathological ageing and more recently in mood disorders, where positive effects on cognitive function have been shown (Hammar et al. 2022; Hoorelbeke and Koster 2017; Woolf et al. 2022). Similarly, positive effects on cognitive function have been observed following AT in patient groups with mood disorders (Falkai et al. 2022; Ren et al. 2024). Although most studies have focused on immediate gains and transfer effects following CCT and AT, fewer have investigated the long‐term effects of such interventions. This is unfortunate, since it is important to understand the durability of training gains and transfer effects in order to optimise training interventions, considering that cognitive dysfunction is commonly observed as a residual symptom in patients with mood disorders (Woolf et al. 2022), as well as in patients with ED (Glise et al. 2020). The few studies addressing long‐term effects of CCT in mood disorders have shown maintained cognitive gains in performance‐based measures but mixed results concerning self‐reported cognitive functioning (Hagen and Stubberud 2021; Hoorelbeke et al. 2021; Myklebost et al. 2023). Long‐term effects of AT on cognitive function require sustained physical activity and appear stronger in populations experiencing cognitive deficits (Hötting et al. 2012; Ludyga et al. 2020). Moreover, symptom reduction in both depression (Woolf et al. 2022) and anxiety (Wang et al. 2023) have been shown after CCT, highlighting the importance of evaluating the effects of CCT in patient groups suffering from long‐term ill‐health.

This study is a follow‐up study as part of a randomized controlled trial, REhabilitation for improved COgnition (RECO), investigating the effects of two add‐on interventions, CCT and AT, for patients diagnosed with ED who participated in a multimodal stress rehabilitation programme (MMR). Previous publications from the project have shown that, immediately following the intervention, CCT led to cognitive improvements on trained and near transfer tasks (Gavelin et al. 2015), whereas AT led to improvements in aerobic capacity and episodic memory (Eskilsson et al. 2017). At 1‐year follow‐up (Gavelin et al. 2018), maintenance of gains on a global cognitive score and a trained updating task were observed in the CCT group, whereas the cognitive and aerobic gains of AT were not maintained, likely because patients did not continue regular physical activity. Both intervention groups showed general improvements in psychological health and work ability, but these were not greater than in the control group.

Building on these findings, the aim of this study was to examine the extended long‐term effects of CCT and AT on cognitive function (test‐based and self‐reported), psychological health, and work ability in patients with prior ED, 4.5‐years after the intervention.

We hypothesised that the CCT group would continue to show maintenance of the previous gains on the global cognitive score and the trained updating task at the extended follow‐up, whereas the AT group would not display extended long‐term effects, as their initial post‐intervention gains were no longer present at the 1‐year follow‐up.

## 2 Methods

### 2.1 Study Design

**FIGURE 1:**
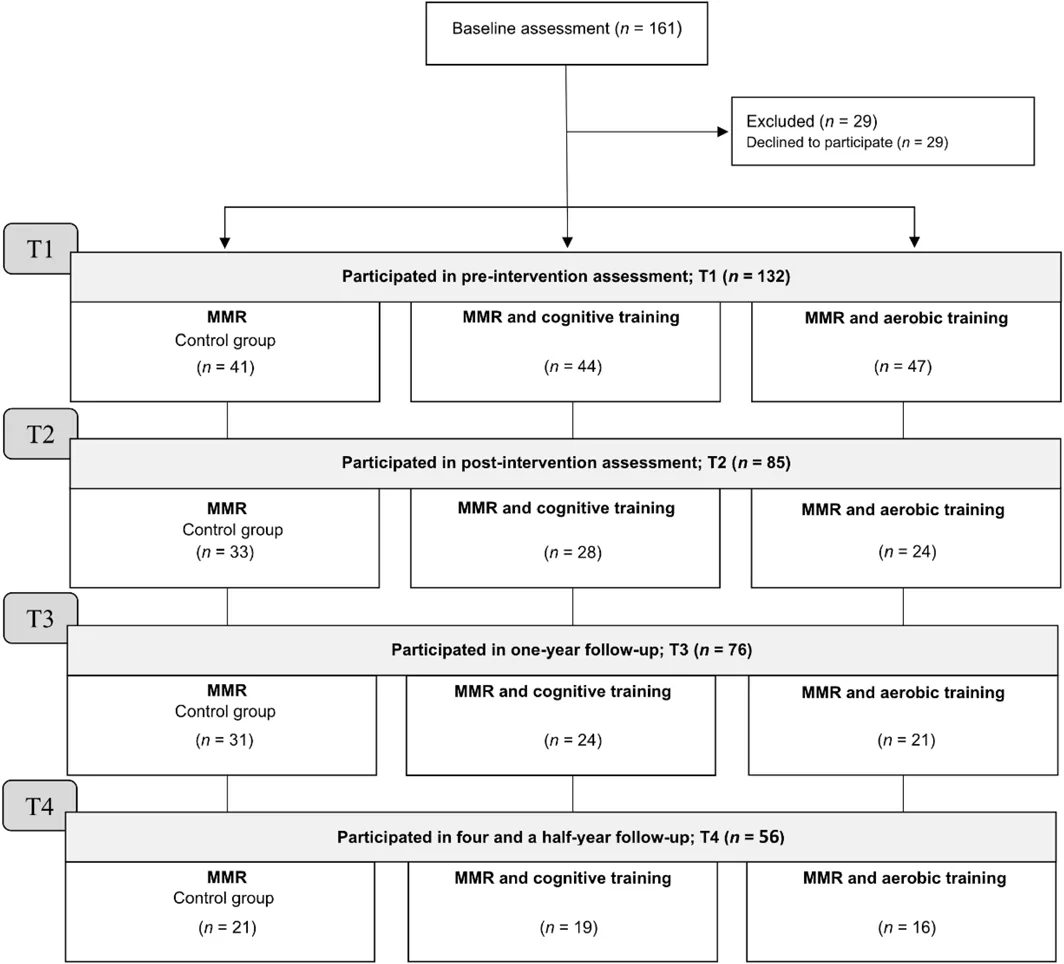
Flowchart of the RECO study.

This study was a follow‐up study as part of a longitudinal randomized controlled trial, REhabilitation for Improved COgnition (RECO). Additional information on the design of the RECO‐project, including a comprehensive description of the randomisation process, blinding and sample size can be found in Gavelin et al. (2018). A flowchart of the RECO‐project is shown in Figure 1.

In this study, the primary outcome was change in cognitive performance across the test‐sessions, measured by a global cognitive score. This composite score was calculated as the mean z‐score of all tests in the cognitive test battery, except for the criterion task trained in the CCT programme. Secondary outcomes included domain‐specific cognitive functioning, self‐reported cognitive function, psychological health (burnout, depression, anxiety, and fatigue), and work ability.

### 2.2 Participants and Procedure

The study was conducted at a specialised stress rehabilitation clinic within a university hospital, where all participants underwent group treatment, a 24‐week multimodal stress rehabilitation programme (MMR) based on cognitive behavioural therapy. Participants were recruited consecutively from April 2010 to June 2013 and screened for eligibility. A total of 161 individuals aged 18–60 diagnosed with ED were recruited. All participants were on sick‐leave due to their illness, while the degree of sick‐leave varied. Inclusion criteria for the study were: (1) confirmed ED according to criteria established by the Swedish National Board of Health and Welfare, (2) 18–60 years of age, (3) current employment, (4) considered by physician and psychologist as suitable for multimodal rehabilitation in group, (5) no known abuse of alcohol or drugs, (6) no need of other treatment or rehabilitation and (7) no participation in other intervention studies. See Appendix 2 for demographics of the participants at baseline. Midway of MMR (at 12 weeks), participants conducted pre‐intervention assessment (T1) consisting of cognitive tests and self‐reported psychological health measures and were then randomly assigned to one of three groups in addition to the multimodal rehabilitation programme; (1) control group, that is, no additional intervention part from MMR (2) CCT and (3) AT (indoor cycling). The additional interventions were conducted over a period of 12 weeks. To evaluate the effects of the interventions, cognitive tests and self‐reported psychological health measures were conducted directly after the intervention ended (T2). At both 1‐year (T3) and 4.5‐year (T4) follow‐up, we contacted participants who had participated at the previous time‐point with a letter with information about the study, followed by a telephone call where they were asked to participate.

This resulted in 132 participants at the pre‐intervention assessment (29 participants declined participation), 85 participants in the post‐intervention assessment, 76 participants in 1‐year follow‐up and 56 participants in the 4.5‐year follow‐up. The final 4.5‐year follow‐up assessment was conducted between June 2015 and May 2018. Participants were not asked why they declined participation at follow‐up due to ethical reasons.

Trained psychologists administered the cognitive tests during each assessment session. The cognitive assessments, with an approximate duration of 2 hours, were typically conducted in a single day, with a 15‐min break. In certain instances, for example due to extensive exhaustion, the testing procedure extended over two separate days. Self‐report questionnaires were completed either on‐site during the testing session or at home and brought to the session.

Participants received financial compensation at each assessment time, with a sum of 1000 SEK (approximately 100 Euros) provided for the T4 assessment. The study followed the guidelines of the Declaration of Helsinki and was approved by the Ethical Review Board. Potential harms, that is, fatigue, discomfort, and worry, were specified in the ethics application and communicated to the participants. Harms were assessed non‐systematically during the neuropsychological testing and at follow‐ups during the intervention period. Participants also had the opportunity to address harms in contact with clinicians in the parallel MMR treatment. Prior to participation, all individuals provided written informed consent.

### 2.3 Interventions

Both interventions were conducted three times a week during a period of 12 weeks. After ending the intervention, no further engagement was required. The training programs are described briefly, as they have been presented in detail elsewhere (Eskilsson et al. 2017; Gavelin et al. 2015).

#### 2.3.1 Computerised Cognitive Training

The CCT programme was in‐house developed and tailored for patients with ED, consisting of tasks addressing cognitive abilities known to be affected in ED. The programme was internet‐based and accessed through a computer. It was approximately 20 minutes long, consisting of six different tasks, two tasks targeting the executive functions shifting and updating respectively, one task targeting episodic memory and one task targeting visuospatial short‐term memory. See Appendix 3 for an overview of the tasks in the training programme. All tasks in the programme except for one were adaptive, meaning that the degree of difficulty on each task was adjusted based on the performance of the patient, theoretically important to keep the training challenging, enhancing the effects of the training period (Lövden et al. 2010). After each completed task, the participants received performance feedback. During the first week of the intervention, the CCT was conducted at the clinic. The remaining sessions were conducted from home with follow‐ups (e‐mail or telephone) with a psychologist providing motivational and, if needed, technical support. At the start of the intervention, follow‐ups were conducted once a week, gradually scaled back during the training period.

#### 2.3.2 Aerobic Training

For AT, the participants engaged in instructor‐led group indoor cycling (‘spinning’), conducted at different training centres of the participants choice. The training sessions were 40 minutes long and the participants were instructed to exercise at 70%–85% of their age‐adjusted maximum heart rate, corresponding to a moderate to vigorous intensity. To monitor this, they wore a chest belt heart monitor (Polar RS800) during the training session. Weekly, a physiotherapist provided written feedback on training intensity and motivational support.

### 2.4 Cognitive Outcomes

At each assessment time, the same cognitive test battery was administered, apart from SRB:1 (a multiple‐choice verbal ability test) that was only conducted at T1, for assessing verbal ability as an indication of premorbid ability. The cognitive test battery consisted of tests assessing executive function, working memory, episodic memory, perceptual speed, and reasoning ability. The test battery has been described in a previous publication (Gavelin et al. 2015); therefore, only a brief overview is presented here.

#### 2.4.1 Executive Function

Three different tests were used to assess executive function. For assessing *updating*, we used the n‐back test. This task was conducted on a computer, where participants were presented with a sequence of single digits (1–9) and asked to decide during the sequence if the digit was the same as the one presented *n* trials ago. Three different n‐back conditions were used, 1‐ 2‐ and 3‐back, where accuracy in the 3‐back condition was used as the target outcome. For assessing *inhibition*, we used the Colour‐word interference test and for assessing *shifting ability*, we used the Trail making test, both from D‐KEFS (Delis et al. 2001) and administered according to the standardized procedures. For outcome measures, inhibition cost and shift cost were calculated by subtracting the baseline performance from the incongruent‐ and mixed conditions, respectively.

#### 2.4.2 Working Memory

Two test conditions were used to assess working memory, Digit span forwards and backwards from WAIS‐R (Wechsler 1981) and Letter‐number sequencing from WAIS III (Wechsler 1997), both administered according to the standard procedures. For outcome measures, the total number of correct responses were used.

#### 2.4.3 Episodic Memory

One test was administered to assess episodic memory, Recall of concrete nouns (Buschke 1973). The test consisted of 18 nouns presented four times for the participants according to the selective reminding procedure, meaning that only those words the participant did not remember were reminded in the next presentation of the list. For outcome measure, the total number of correctly recalled words at all four trials was used.

#### 2.4.4 Perceptual Speed

One test was administered for assessing perceptual speed, Digit symbol from WAIS‐R (Wechsler 1981). The test was administered according to the standard procedure and for outcome measure the total number of correct drawn symbols was used.

#### 2.4.5 Reasoning Ability

One test was administered for assessing reasoning ability, Raven's advanced progressive matrices (Raven et al. 1998). The administration of the test was adjusted from the standard procedure to facilitate repeated measures and control the length of the total battery. Therefore, the 36 matrices were divided in half, where odd numbers made up one battery and even numbers made up the other battery. Ten minutes were given to solve the matrices. Total number of correct solved matrices during these 10 minutes were used as our outcome measure.

#### 2.4.6 Cognitive Training Criterion Task

The criterion task, Letter memory running span (Miyake et al. 2000) was addressing the executive function updating. The task was used in the training programme as well as an outcome measure. The task was administered on a computer, and the participants were presented with a sequence of single letters (A−D). At the end of the sequence, the task for the participants was to recall the four last seen letters in the correct order. Ten letter‐sequences were presented for the participant, and the outcome measure was the number of fully correct sequences.

#### 2.4.7 Self‐Reported Memory Problems

Self‐reported measures of memory problems were collected at T1, T2 and T4. Due to an administrative error, data is partially missing at T2. Also, no data was collected at T3 due to changes in clinical routines. To assess memory problems, two self‐report questionnaires were used: the Prospective and Retrospective Memory Questionnaire (PRMQ) (Crawford et al. 2003) and the 6‐item questionnaire on everyday memory problems (6‐QEMP). The PRMQ consists of 16 items (e.g., ‘Do you decide to do something in a few minutes' time and then forget to do it?’) rated on a 5‐point Likert scale from 1 (never) to 5 (very often). A total score was computed (range 16–80) with higher scores indicating more memory problems. The 6‐QEMP consists of six‐items and was developed specifically to assess cognitive problems related to stress (Österberg et al. 2009). It includes general memory‐related questions (e.g., ‘Does anybody close to you [family, friends] think you have a poor memory?’), as well as items specifically addressing memory problems in relation to stress (e.g., ‘How is your memory today, in comparison with when you had the most problems with your stress‐related illness?’). All items are rated on a 5‐point Likert scale with various response labels. Total score (range 6–30) was used as outcome measure and higher values indicate more memory problems.

### 2.5 Psychological Health and Work Ability Outcomes

Self‐reported measures of psychological health were conducted at each assessment time. The variables investigated were level of burnout, depression, anxiety, and fatigue.

#### 2.5.1 Burnout

Shirom‐Melamed Burnout Questionnaire (SMBQ) was used to assess level of burnout (Melamed et al. 1992). While SMBQ is not developed for a clinical population, the association between measures developed for ED specifically and SMBQ is high (Persson et al. 2017). SMBQ consists of 22 items (e.g., ‘I feel burned out’), on a 7‐point Likert scale ranging from 1 (almost never) to 7 (almost always) and the mean value of the items (range 1–7) was used as an outcome measure, where higher values indicate more severe burnout.

#### 2.5.2 Depression

The Hospital Anxiety and Depression Scale (HADS) was used to assess level of depression (Zigmond and Snaith 1983). The questionnaire consists of 14 items rated on a 4‐point Likert scale ranging from 0 to 3. Seven of the 14 items in the questionnaire targets depression (e.g., ‘I look forward with enjoyment to things’). A total score was computed for the items on depression (range 0–21) with higher scores reflecting greater levels of depression.

#### 2.5.3 Anxiety

HADS was used to assess level of anxiety. Seven of the 14 items in the questionnaire targets anxiety (e.g., ‘I feel tense or wound up’), on a 4‐point Likert scale and the total score of the seven anxiety‐items (range 0–21) were used as an outcome measure where higher score indicates more anxiety.

#### 2.5.4 Fatigue

The Checklist Individual Strength (CIS) was used to assess fatigue (Beurskens et al. 2000). The questionnaire comprises 20 items (e.g., ‘I feel like doing all kinds of nice things’), rated on a 7‐point Likert scale ranging from 1 (yes, i.e., true) to 7 (no, i.e. not true). A total score was computed (range 20–140) with higher scores reflecting greater levels of fatigue.

#### 2.5.5 Work Ability

The Work Ability Index (WAI) was used to assess work ability (de Zwart et al. 2002). The questionnaire comprises 10 items (e.g., ‘How do you rate your current work ability with respect to the physical demands of your work?’), rated on various Likert scales. Total score (range 7–49) was used as outcome measure and higher values indicate better work ability.

### 2.6 Statistical Analysis

Statistical analyses were performed using SPSS (IBM Corp 2020) for dropout analysis and R (R Core Team 2024) for linear mixed models. Drop‐out analyses were conducted using independent samples *t*‐test for continuous variables and Pearsons Chi‐square test for categorical variables. Drop‐out analyses on baseline characteristics (age, sex, educational level, and verbal ability) and self‐reported psychological health at T1, T2 and T3 (burnout, anxiety, depression, fatigue, and work ability) were performed. Analyses were conducted separately for each group, except for baseline characteristics where the whole sample were compared in addition to the separate group analyses.

Prior to the analysis of the main outcomes, all cognitive test scores were *z*‐transformed using baseline mean and standard deviation, with a higher score indicating a better performance. Missing data in SMBQ at T4 (single items for five participants) were handled by expectation–maximisation imputation. Missing data for WAI at T4 could not be imputed, as the missing items were context‐dependent and forward‐looking, precluding reliable estimations (e.g., personal prognosis of work ability 2 years from now). All analyses (except for the complete‐case sensitivity analysis) were conducted on all available data. Number of participants varied for different variables, due to missing data. For information on *N*s for each variable, see Tables 1 and 2, while matrices illustrating the proportion of available data for each variable are presented in Supporting Information S1.

**TABLE 1:** Group means and standard deviations on the cognitive variables at each assessment‐time.

Variable	Control group				Computerised cognitive training				Aerobic training			
	T1	T2	T3	T4	T1	T2	T3	T4	T1	T2	T3	T4
Global cognitive	0.12 (0.66)	0.33 (0.53)	0.38 (0.55)	0.51 (0.43)	−0.06 (0.63)	0.25 (0.68)	0.40 (0.81)	0.43 (0.72)	−0.08 (0.53)	0.18 (0.55)	0.29 (0.59)	0.25 (0.44)
Score	*n* = 36	*n* = 30	*n* = 26	*n* = 21	*n* = 39	*n* = 27	*n* = 21	*n* = 18	*n* = 43	*n* = 23	*n* = 20	*n* = 16
Executive function												
3‐back	22.50 (6.97)	25.56 (5.59)	25.46 (6.19)	25.90 (4.88)	20.33 (6.22)	24.54 (7.65)	25.43 (8.58)	25.22 (8.29)	19.70 (8.30)	23.61 (8.03)	25.95 (6.52)	25.50 (6.56)
	*n* = 38	*n* = 32	*n* = 26	*n* = 21	*n* = 39	*n* = 28	*n* = 21	*n* = 18	*n* = 43	*n* = 23	*n* = 21	*n* = 16
Shift cost	44.60 (25.36)	38.22 (20.75)	43.35 (14.11)	33.48 (15.26)	53.29 (27.63)	48.64 (33.01)	41.39 (17.92)	37.68 (16.26)	48.07 (19.81)	42.54 (20.94)	42.57 (25.05)	41.88 (16.89)
	*n* = 40	*n* = 32	*n* = 26	*n* = 21	*n* = 42	*n* = 28	*n* = 23	*n* = 19	*n* = 46	*n* = 24	*n* = 21	*n* = 16
Inhibition cost	29.00 (14.41)	23.47 (7.98)	23.15 (8.26)	22.05 (5.82)	29.93 (9.02)	25.46 (8.33)	31.04 (16.54)	26.11 (7.02)	29.87 (11.56)	28.67 (10.95)	26.29 (10.16)	25.88 (9.52)
	*n* = 40	*n* = 32	*n* = 26	*n* = 21	*n* = 42	*n* = 28	*n* = 23	*n* = 19	*n* = 46	*n* = 24	*n* = 21	*n* = 16
Working memory												
Digit span forwards	7.22 (2.11)	7.19 (1.69)	6.96 (1.66)	7.29 (1.55)	6.83 (2.00)	7.36 (2.13)	7.70 (2.16)	7.42 (2.57)	6.70 (1.95)	6.71 (2.33)	6.48 (2.29)	7.00 (2.31)
	*n* = 40	*n* = 32	*n* = 26	*n* = 21	*n* = 42	*n* = 28	*n* = 23	*n* = 19	*n* = 46	*n* = 24	*n* = 21	*n* = 16
Digit span backwards	6.20 (1.76)	6.65 (1.70)	6.88 (1.95)	7.33 (1.68)	6.74 (2.30)	7.07 (2.11)	7.52 (2.84)	7.42 (2.52)	6.46 (1.93)	6.42 (2.10)	6.43 (2.66)	6.75 (1.81)
	*n* = 40	*n* = 31	*n* = 26	*n* = 21	*n* = 42	*n* = 28	*n* = 23	*n* = 19	*n* = 46	*n* = 24	*n* = 21	*n* = 16
Letter‐number‐sequencing	10.31 (2.02)	10.38 (2.43)	10.42 (2.39)	11.43 (2.06)	9.88 (2.93)	10.22 (2.75)	10.90 (2.79)	11.21 (2.90)	9.22 (2.25)	10.29 (2.69)	10.30 (2.75)	10.44 (2.50)
	*n* = 39	*n* = 32	*n* = 26	*n* = 21	*n* = 40	*n* = 27	*n* = 21	*n* = 19	*n* = 45	*n* = 24	*n* = 20	*n* = 16
Episodic memory												
Recall of concrete nouns	52.46 (9.83)	53.58 (11.19)	55.92 (12.03)	56.48 (10.25)	50.50 (11.34)	54.71 (13.37)	56.57 (11.57)	58.79 (11.82)	50.11 (8.21)	55.67 (6.91)	55.30 (7.37)	55.38 (7.71)
	*n* = 37	*n* = 31	*n* = 26	*n* = 21	*n* = 40	*n* = 28	*n* = 21	*n* = 19	*n* = 44	*n* = 24	*n* = 20	*n* = 16
Perceptual speed												
Digit symbol	54.83 (11.88)	59.62 (13.42)	60.27 (12.66)	59.67 (9.98)	50.93 (9.07)	57.39 (12.08)	59.91 (14.21)	57.32 (11.29)	51.72 (9.63)	56.21 (9.98)	57.33 (9.37)	54.19 (12.16)
	*n* = 40	*n* = 32	*n* = 26	*n* = 21	*n* = 42	*n* = 28	*n* = 23	*n* = 19	*n* = 46	*n* = 24	*n* = 21	*n* = 16
Reasoning ability												
Raven's matrices	6.90 (2.70)	6.28 (2.73)	7.65 (3.14)	7.14 (2.50)	6.60 (3.01)	7.29 (2.81)	7.71 (3.72)	6.74 (3.35)	7.48 (2.81)	6.71 (2.76)	7.50 (3.07)	6.75 (2.21)
	*n* = 39	*n* = 32	*n* = 26	*n* = 21	*n* = 40	*n* = 28	*n* = 21	*n* = 19	*n* = 44	*n* = 24	*n* = 20	*n* = 16
Cognitive training criterion task												
Letter memory running span	2.40 (1.79)	2.66 (1.62)	2.62 (1.55)	2.90 (1.87)	2.07 (1.59)	4.36 (2.26)	4.09 (1.78)	4.00 (2.00)	2.34 (1.64)	1.96 (1.65)	2.95 (1.72)	3.13 (1.67)
	*n* = 40	*n* = 32	*n* = 26	*n* = 21	*n* = 41	*n* = 28	*n* = 23	*n* = 19	*n* = 44	*n* = 24	*n* = 21	*n* = 16

*Note:* T1: pre‐intervention; T2: post‐intervention; T3: 1‐year follow‐up; T4: 4.5‐year follow‐up. Group means and standard deviations of raw scores are shown for all variables except global cognition (*z*‐transformed). Higher scores indicate better performance.

**TABLE 2:** Group means and standard deviations on psychological variables, self‐reported cognitive function and work ability at each assessment‐time.

Variable	Control group				Cognitive training group				Aerobic training group			
	T1	T2	T3	T4	T1	T2	T3	T4	T1	T2	T3	T4
SMBQ	4.78 (1.03)	4.34 (1.07)	3.94 (1.25)	4.57 (1.16)	4.98 (0.88)	4.20 (1.06)	3.95 (1.02)	3.61 (1.01)	4.87 (0.95)	4.05 (1.22)	4.05 (1.28)	3.30 (1.38)
	*n* = 38	*n* = 40	*n* = 40	*n* = 21	*n* = 38	*n* = 44	*n* = 40	*n* = 19	*n* = 43	*n* = 46	*n* = 45	*n* = 16
HADS												
Depression	7.26 (3.48)	6.05 (4.14)	5.25 (4.12)	5.52 (3.84)	6.76 (2.70)	4.73 (2.82)	4.58 (2.68)	3.63 (2.93)	6.81 (3.79)	4.93 (3.84)	4.82 (3.55)	3.13 (2.58)
	*n* = 38	*n* = 40	*n* = 40	*n* = 21	*n* = 38	*n* = 44	*n* = 40	*n* = 19	*n* = 43	*n* = 46	*n* = 45	*n* = 16
Anxiety	9.41 (3.82)	8.40 (3.67)	7.18 (4.30)	8.81 (4.76)	10.47 (3.03)	7.75 (3.15)	7.70 (4.03)	7.58 (3.31)	9.19 (3.83)	6.87 (3.40)	7.67 (4.73)	5.44 (4.38)
	*n* = 38	*n* = 40	*n* = 40	*n* = 21	*n* = 38	*n* = 44	*n* = 40	*n* = 19	*n* = 43	*n* = 46	*n* = 45	*n* = 16
CIS	96.02 (19.64)	86.93 (22.58)	79.70 (24.43)	86.00 (24.62)	95.83 (17.87)	85.01 (21.06)	79.88 (20.31)	67.42 (19.74)	94.24 (19.45)	79.54 (21.90)	82.11 (26.60)	66.59 (26.49)
	*n* = 38	*n* = 40	*n* = 40	*n* = 21	*n* = 38	*n* = 43	*n* = 40	*n* = 19	*n* = 43	*n* = 46	*n* = 45	*n* = 16
PRMQ^a^	49.44 (10.96)	47.53 (10.49)	—	49.40 (12.72)	49.82 (11.67)	42.77 (11.11)	—	42.00 (11.21)	50.65 (10.70)	45.73 (6.60)	—	43.47 (10.41)
	*n* = 36	*n* = 17		*n* = 21	*n* = 39	*n* = 26		*n* = 18	*n* = 37	*n* = 15		*n* = 15
6‐QEMP^a^	20.58 (3.79)	20.82 (3.76)	—	20.86 (4.29)	21.61 (3.51)	18.00 (4.24)	—	17.68 (4.63)	21.47 (3.41)	19.23 (2.83)	—	17.20 (2.86)
	*n* = 36	*n* = 17		*n* = 14	*n* = 38	*n* = 28		*n* = 19	*n* = 38	*n* = 13		*n* = 15
WAI	27.41 (6.94)	30.08 (6.79)	33.40 (6.09)	32.21 (7.37)	26.29 (6.91)	29.68 (6.77)	34.02 (7.27)	34.79 (7.97)	26.71 (6.29)	31.77 (5.34)	31.83 (9.25)	38.77 (4.80)
	*n* = 38	*n* = 33	*n* = 28	*n* = 19	*n* = 38	*n* = 28	*n* = 23	*n* = 19	*n* = 43	*n* = 24	*n* = 19	*n* = 15

*Note:* T1: pre intervention; T2: post intervention; T3: 1‐year follow‐up, T4: 4.5‐year follow‐up. For all variables except for WAI, lower score indicates less symptoms. For WAI, higher scores indicates better work ability. —indicate no data available for T3.

^a^ Data is partially missing at T2.

The lme4 package (Bates et al. 2015) was used for the linear mixed effect analyses. To obtain *p*‐values, the lmerTest package was used with the Satterthwaite approximation for degrees of freedom (Kuznetsova et al. 2017). See Supporting Information S2 for selected R code illustrating the main steps of the analyses. In linear mixed models, missing data is assumed to be missing at random. The model includes all available data.

The effects of the intervention on cognitive function, psychological health, work ability and self‐reported cognitive function were analysed using the same pre‐specified linear mixed‐effects model: *Outcome ∼* Group × Time + (1|Participant), fitted with restricted maximum likelihood estimation. The models included fixed effects for group, time, and their interaction and a random intercept at participant level. Time was modelled as a categorical variable due to irregular spacing between assessment times. Analyses were performed according to the intention‐to‐treat principle. All three groups (MMR, CCT and AT) and all four assessment times (T1−T4) were included in the model, ensuring that the full longitudinal data structure contributed to the estimation of the effects. As the model was pre‐specified, no model comparisons were conducted.

Our main interest was the Group × Time interaction from T1 to T4, reflecting the change from pre‐intervention to 4.5‐year follow‐up for the intervention groups relative to the control group. Hence, the reference levels were the control group for group and T1 for time. Results from the full models are included in the Supporting Information [Link], [Link], [Link], [Link], [Link], [Link]. Significant interaction effects from T1 to T4 were followed by post‐hoc pairwise comparisons of the change between T3 and T4 within each group, to examine whether the outcomes from the 1‐year follow‐up were maintained or had changed further. These comparisons were based on the estimated marginal means from the model, using the emmeans package (Lenth 2025). Accordingly, in this study, extended long‐term effects were defined as a significant Group × Time interaction from T1 to T4, indicating differences in change between the intervention groups and the control group over the full study period. Follow‐up comparisons between T3 and T4 explored whether these effects represented maintenance of previous gains (i.e., stability in outcomes between T3 and T4) or potential new changes emerging between the 1‐year and 4.5‐year follow‐up.

To assess the robustness of the findings, sensitivity analyses were conducted. First, a complete‐case analysis was performed by fitting the linear mixed model to participants with data at all assessment time points. Second, a pattern‐mixture model (PMM) was applied to evaluate the potential impact of missing data, classifying participants as completers or non‐completers based on their missing‐data pattern at the final assessment (T4). Due to small subgroup sizes, more detailed classifications were not feasible. The primary parameter of interest was the Group × Time × Pattern interaction; however, as non‐completers contributed no data at T4, inferences were restricted to time points with data from both groups. In addition, *p*‐values were adjusted for multiple comparisons using the Benjamini–Hochberg (BH) procedure to control the false discovery rate, as no such correction was applied in the primary analyses. BH corrections were applied within two families of outcomes, separating cognitive tests and self‐reported measures.

Figures were generated using the package ggplot2 (Wickham 2016).

## 3 Results

### 3.1 Characteristics at T1 and Drop‐Out Analyses

As can be seen in Figure 1, 49% in the MMR group, 45% of the CCT group and 34% in the AT group returned to the 4.5‐year assessment relative to the pre‐test participation. In the whole sample, there were no significant differences at T1 between participants who completed the 4.5‐year follow‐up (T4) and non‐completers (i.e., dropouts) with respect to age, sex or education. However, completers had significantly higher verbal ability compared to non‐completers. Additional dropout analyses conducted separately for each group on burnout, depression, anxiety and fatigue showed that, within the control group, completers reported higher levels of fatigue at T2 and T3 compared to dropouts. Further, non‐completers within the CCT group reported higher burnout and fatigue at T2 and T3. They also reported statistically significantly higher levels of depression at T3. No other significant differences between completers and non‐completers were observed within either group at T1, T2 or T3. Results from the drop‐out analyses are presented in Supporting Information S3.

### 3.2 Cognitive Function

#### 3.2.1 Cognitive Test Performance

Table 1 presents the group means and standard deviations for the cognitive variables at all assessment times. Results from the Group × Time interaction analysis from T1 to T4 are presented in Table 3 (see Supporting Information S4 for the results from the full models). Pairwise comparisons between T3 and T4, conducted for variables with significant results in the main analysis, are presented in Table 4.

**TABLE 3:** Training‐related effects on cognitive variables.

Variable	Group (ref. control group)	T1 to T4		
		*β* (95% CI)	*p*‐value	*d*
Global cognitive score	Cognitive training	0.25 (0.06, 0.44)	0.011	0.41
	Aerobic training	0.08 (−0.12, 0.27)	0.438	0.13
Executive function	Cognitive training	0.12 (−0.20, 0.43)	0.459	0.17
	Aerobic training	−0.02 (−0.34, 0.30)	0.894	0.03
Working memory	Cognitive training	0.16 (−0.18, 0.49)	0.358	0.19
	Aerobic training	−0.01 (−0.36, 0.33)	0.935	−0.01
Episodic memory	Cognitive training	0.68 (0.21, 1.15)	0.005	0.68
	Aerobic training	0.39 (−0.10, 0.88)	0.116	0.39
Perceptual speed	Cognitive training	0.26 (−0.14, 0.66)	0.195	0.26
	Aerobic training	0.25 (−0.17, 0.66)	0.247	0.25
Reasoning ability	Cognitive training	0.15 (−0.39, 0.69)	0.582	0.15
	Aerobic training	−0.06 (−0.62, 0.49)	0.821	−0.06
Cognitive training criterion task	Cognitive training	0.77 (0.13, 1.42)	0.019	0.77
	Aerobic training	0.25 (−0.42, 0.92)	0.457	0.25

*Note:* Estimates represent the average difference in change across sessions between intervention group and control group, based on the *z*‐transformed test scores. For all variables, a positive value indicates improved performance. Cohen's *d* is calculated as the difference in mean change across sessions between intervention group and control group, divided by the baseline standard deviation. T1: pre intervention; T4: 4.5‐year follow‐up.

**TABLE 4:** Pairwise comparisons between T3 and T4 within each group based on the fitted mixed‐effects models.

Variable	Group	Estimated mean difference (T4‐T3)	
		Estimate (95% CI)	*p‐*value
Global cognitive score	Control group	−0.08 (−0.21, 0.06)	0.249
	Cognitive training	−0.03 (−0.17, 0.12)	0.729
	Aerobic training	−0.06 (−0.21, 0.09)	0.434
Episodic memory	Control group	−0.04 (−0.38, 0.31)	0.831
	Cognitive training	0.35 (−0.02, 0.71)	0.063
	Aerobic training	0.03 (−0.35, 0.42)	0.878
Cognitive training criterion task	Control group	−0.04 (−0.52, 0.44)	0.870
	Cognitive training	−0.13 (−0.63, 0.36)	0.595
	Aerobic training	0.12 (−0.42, 0.65)	0.664

*Note:* Estimates represent the difference between the estimated marginal means at T3 and T4. A negative value indicates a decline in performance from T3 to T4, whereas a positive value indicates an improvement from T3 to T4.

For the CCT group, the Group × Time interaction for the global cognitive score was significant (*p* = 0.011, Cohen's *d* = 0.41), indicating greater improvement than the control group from T1 to T4. No significant interaction was found for the AT group (*p* = 0.438, Cohen's *d* = 0.13). Post hoc pairwise comparisons showed no significant changes between T3 and T4 in either group (CCT: *p* = 0.729; control: *p* = 0.249; AT: *p* = 0.434). Moreover, a significant Group × Time interaction favouring the CCT group was found for episodic memory (*p* = 0.005, Cohen's *d* = 0.68), whereas the interaction was not significant for the AT group (*p* = 0.116, Cohen's *d* = 0.39). Post hoc pairwise comparisons showed no significant changes from T3 to T4 in either group (CCT: *p* = 0.063; control group: *p* = 0.831; AT: *p* = 0.878). Finally, a statistically significant Group × Time interaction was found for the CCT group on the criterion updating task (*p* = 0.019, Cohen's *d* = 0.77), showing a greater improvement from T1 to T4 compared to the control group. For the AT group, the Group × Time interaction was not significant (*p* = 0.457, Cohen's *d* = 0.25). Post hoc pairwise comparisons showed no significant changes from T3 to T4 in either group (CCT: *p* = 0.595; control group: *p* = 0.870; AT: *p* = 0.664). No statistically significant interaction effects were found for executive function, working memory, perceptual speed or reasoning ability (Table 3).

### 3.3 Self‐Reported Memory Problems

Group means and standard deviations for self‐reported memory problems are presented in Table 2. Results from the Group × Time interaction analysis from T1 to T4 are presented in Table 5 (see Supporting Information S5 for the results from the full models). Due to missing data, no pairwise comparisons between T3 and T4 were conducted.

**TABLE 5:** Training‐related effects on psychological variables, self‐reported cognitive function and work ability.

Variable	Group (ref. control group)	T1 to T4		
		Estimate (95% CI)	*p*‐value	*d*
SMBQ	Cognitive training	−0.69 (−1.27, −0.10)	0.022	−0.73
	Aerobic training	−1.04 (−1.65, −0.44)	< 0.001	−1.09
HADS				
Depression	Cognitive training	−0.50 (−2.35, 1.34)	0.592	−0.15
	Aerobic training	−1.17 (−3.07, 0.73)	0.228	−0.35
Anxiety	Cognitive training	−1.61 (−3.70, 0.48)	0.130	−0.32
	Aerobic training	−2.59 (−4.74, −0.44)	0.018	−0.72
CIS	Cognitive training	−7.76 (−20.00, 4.50)	0.214	−0.41
	Aerobic training	−8.84 (−21.50, 3.78)	0.169	−0.47
PRMQ	Cognitive training	−3.07 (−8.58, 2.44)	0.272	−0.28
	Aerobic training	−6.47 (−12.30, −0.64)	0.030	−0.59
6‐QEMP	Cognitive training	−2.77 (−4.92, −0.61)	0.012	−0.78
	Aerobic training	−4.20 (−6.47, −1.93)	< 0.001	−1.18
WAI	Cognitive training	2.94 (−0.99, 6.86)	0.142	0.40
	Aerobic training	6.74 (2.62, 10.90)	0.001	0.91

*Note:* Estimates represent the average difference in change across sessions between intervention group and MMR group, based on the raw scores. For all variables except for WAI, a negative value indicates a decrease in symptoms. For WAI, a positive value indicates higher work ability. Cohen's *d* is calculated as the difference in mean change across sessions between intervention group and MMR group, divided by the baseline standard deviation. T1: pre intervention; T4: 4.5‐year follow‐up.

For self‐reported memory problems measured with 6‐QEMP, there was a significant Group × Time interaction for the CCT group (*p* = 0.012, Cohen's *d* = −0.78), and the AT group (*p* = < 0.001, Cohen's *d* = −1.18), showing that both intervention groups improved more from T1 to T4 than the control group. For PRMQ, there was no significant Group × Time interaction for the CCT group (*p* = 0.272, Cohen's *d* = −0.28) but a significant difference in average change across time from T1 to T4 between the AT group and the control group (*p* = 0.030, Cohen's *d* = −0.59).

#### 3.3.1 Sensitivity Analysis

The mixed model analysis conducted on the complete‐case population (results not shown) yielded results consistent with the main analysis for both CCT and AT in all the cognitive tests, as well as for self‐rated cognitive function measured with 6‐QEMP, suggesting results were robust to attrition. For PRMQ, however, the significant interaction observed in the AT group was not replicated, suggesting the latter finding should be interpreted with caution. The complete case analysis for the CCT group for PRMQ was not significant, in line with the main analysis. The PMM analyses showed no significant Group × Time × Pattern interaction effects at T2 or T3 for either cognitive outcomes or self‐rated cognitive function, indicating similar trajectories for completers and non‐completers prior to the final assessment. After BH correction, no cognitive outcomes met the adjusted significance threshold (previously significant effects were attenuated to *p* = 0.071–0.089), see Supporting Information S6. For self‐reported measures, 6‐QEMP remained significant for both CCT and AT, whereas PRMQ for AT did not reach the adjusted significance threshold.

#### 3.3.2 Summary of Findings

**FIGURE 2:**
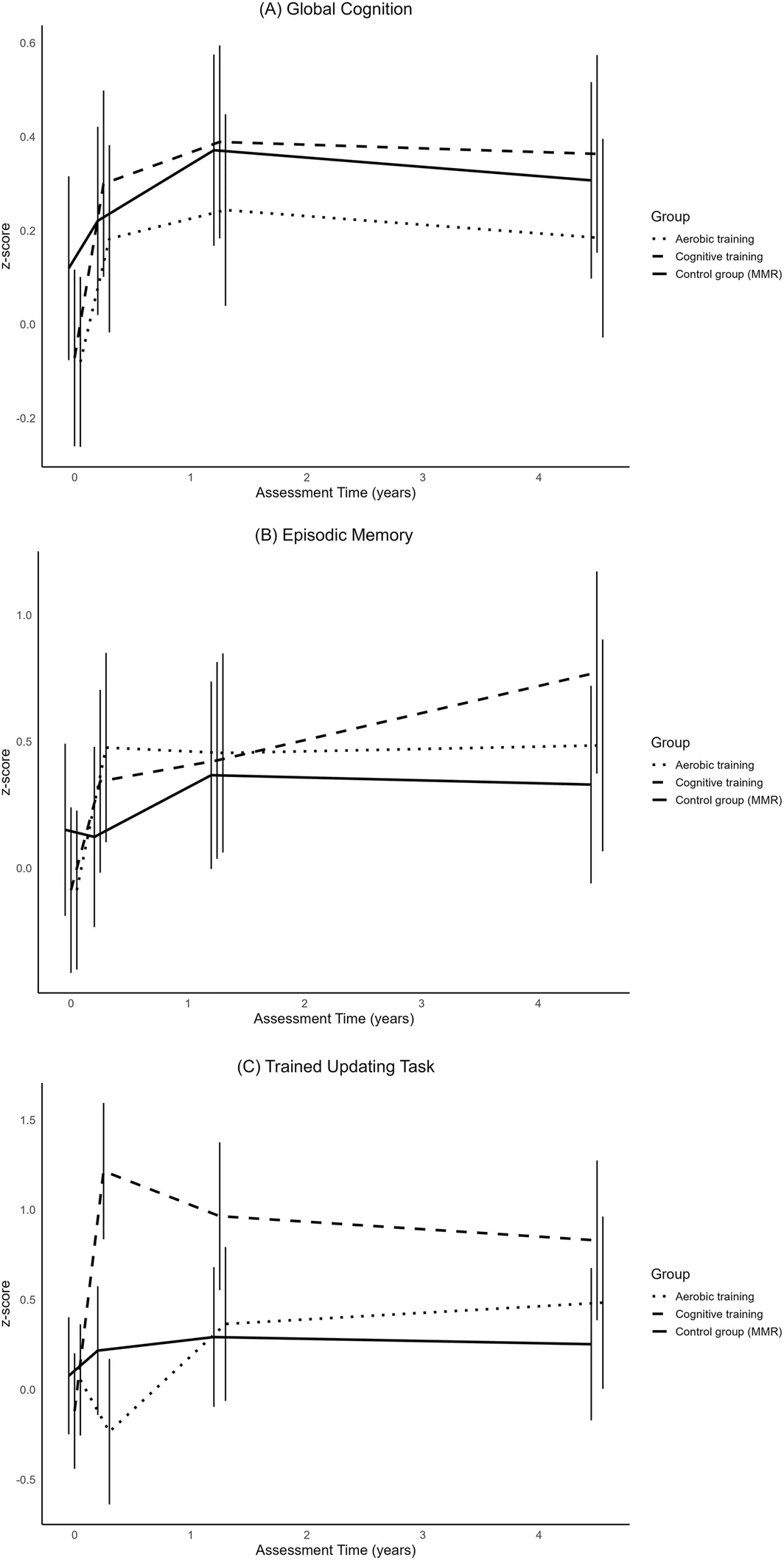
Estimated means over time for global cognition, episodic memory and trained updating task. Changes across time for each group based on the estimated means from the model in (A) performance on the global cognitive score, based on the mean z‐score of nine cognitive tests covering the domains executive function, working memory, episodic memory, processing speed, and reasoning ability; (B) performance in episodic memory and; (C) performance on the cognitive training criterion updating task. For all variables in the figure, higher scores indicate better performance. Error bars indicate confidence interval (CI).

Extended long‐term effects on cognitive test performance were observed in the CCT group on three out of seven outcomes: the global cognitive score, episodic memory and the trained updating task. These effects represented greater improvements from pretest to 4.5‐year follow‐up compared to the control group, and follow‐up comparisons showed no within‐group changes between T3 and T4, indicating maintained performance from 1‐year to 4.5‐year follow‐up. See Figure 2 for a visual presentation of significant results. For self‐reported memory problems, extended long‐term effects favouring both CCT and AT over the control group were found on one of two measures of self‐reported memory (6‐QEMP).

### 3.4 Psychological Health and Work Ability

Table 2 shows the group means and standard deviations for the psychological variables and work ability at each assessment time. Results from the Group × Time interaction analysis from T1 to T4 are presented in Table 5 (see Supporting Information S5 for the results from the full models). In Table 6, pairwise comparisons between T3 and T4, conducted on the variables with significant results in the main analysis, are presented.

**TABLE 6:** Pairwise comparisons for self‐reported psychological health and work ability between T3 and T4 within each group based on the fitted mixed‐effects models.

Variable	Group	Estimated mean difference (T4‐T3)	
		Estimate (95% CI)	*p*‐value
Burnout	Control group	0.41 (0.01, 0.82)	0.046
	Cognitive training	−0.08 (−0.50, 0.35)	0.719
	Aerobic training	−0.69 (−1.14, −0.25)	0.002
Anxiety	Control group	1.31 (−0.16, 2.77)	0.080
	Cognitive training	−0.01 (−1.54, 1.53)	0.993
	Aerobic training	−2.21 (−3.82, −0.60)	0.007
Work ability	Control group	−0.56 (−3.47, 2.34)	0.702
	Cognitive training	0.28 (−2.67, 3.24)	0.850
	Aerobic training	7.41 (4.10, 10.71)	< 0.001

*Note:* Estimates represent the difference between the estimated marginal means at T4 and T3. A negative value indicates reduced symptoms at T4 compared to T3 for burnout and anxiety, and a positive value indicates increased symptoms. For work ability, a positive value indicates higher work ability at T4 compared to T3.

For the psychological variables, there was a statistically significant Group × Time interaction for burnout in both the CCT group (*p* = 0.022, Cohen's *d* = −0.73) and the AT group (*p* < 0.001, Cohen's *d* = −1.09), indicating greater reduction in burnout symptoms from T1 to T4 compared to the control group. Post hoc pairwise comparisons from T3 to T4 showed no significant change within the CCT group (*p* = 0.719), a significant deterioration within the control group (*p* = 0.046) and a significant improvement within the AT group (*p* = 0.002). For anxiety, the Group × Time interaction was not significant for the CCT group (*p* = 0.130, Cohen's *d* = −0.32), but significant for the AT group (*p* = 0.018, Cohen's *d* = −0.72), showing greater improvement from T1 to T4 compared to the control group. Post hoc pairwise comparisons showed no significant changes from T3 to T4 in either the CCT group (*p* = 0.993) or the control group (*p* = 0.080), but a significant improvement within the AT group (*p* = 0.007). No significant interaction effects were found for depression or fatigue (Table 5).

For self‐reported work ability, the Group × Time interaction was not significant for the CCT group (*p* = 0.142, Cohen's *d* = 0.40), but a significant interaction was found for the AT group (*p* = 0.001, Cohen's *d* = 0.91), showing greater improvement from T1 to T4 compared to the control group. Post hoc pairwise comparisons showed no significant changes from T3 to T4 in either the CCT group (*p* = 0.702) or the control group (*p* = 0.850), but a significant improvement in the AT group (*p* = < 0.001).

#### 3.4.1 Sensitivity Analysis

Mixed‐model analysis on the complete case population revealed the same pattern of significant and non‐significant results as the main analysis for burnout, depression, anxiety, fatigue and work ability in both the CCT group and the AT group. The PMM analyses showed significant Group × Time × Pattern interaction effects in the CCT group for WAI and depression at T3, and for CIS and SMBQ at both T2 and T3, indicating that trajectories differed between completers and non‐completers for these outcomes. For all significant interaction effects, non‐completers showed less favourable trajectories over time compared to completers in the CCT group. In contrast, no significant Group × Time × Pattern interactions were observed for the AT group at either T2 or T3. After BH correction, effects for AT remained significant for SMBQ and WAI, whereas effects for SMBQ in CCT and anxiety in AT did not reach the adjusted significance threshold (both *p* = 0.052), see Supporting Information S6.

#### 3.4.2 Summary of Findings

**FIGURE 3:**
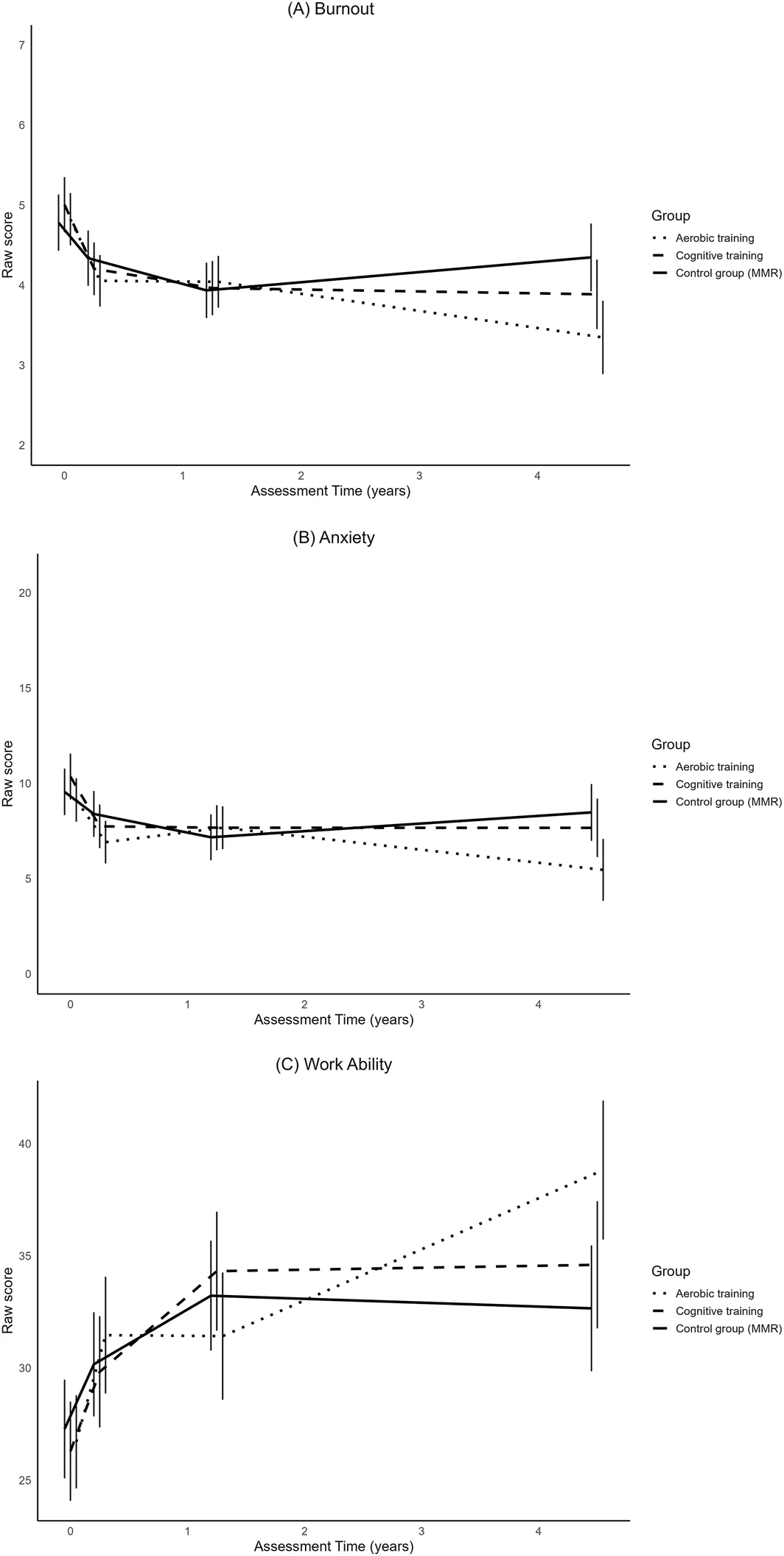
Estimated means over time for burnout, anxiety, and work ability. Changes across time for each group based on the estimated means from the model in (A) burnout, (B) anxiety and (C) work Ability. For burnout and anxiety, lower scores indicate fewer symptoms. For work ability, higher scores indicate better work ability. Error bars indicate confidence intervals (CI).

Extended long‐term effects were observed for burnout in both the CCT group and the AT group, with additional effects on anxiety and work ability in the AT group. These effects represented greater improvements from pretest to 4.5‐year follow‐up compared to the control group. Follow‐up comparisons revealed that between the 1‐year and 4.5‐year assessments, burnout, anxiety and work ability improved in the AT group, while burnout levels increased in the control group and were stable in the CCT group. See Figure 3 for a visual presentation of the significant results for psychological health and work ability.

## 4 Discussion

The aim of this study was to examine the extended long‐term effects, 4.5 years post‐intervention of two add‐on interventions (CCT and AT) to that of MMR with no additional intervention, on cognitive function (test‐based and self‐reported), psychological health, and work ability in patients with prior ED. The main finding of the study was the extended long‐term effects on cognitive performance observed in the CCT group, seen in the primary outcome measure of global cognition, as well as in the criterion updating task and episodic memory, compared to MMR. The addition of AT did not yield any extended long‐term effects on cognitive function. Both intervention groups showed a greater reduction in self‐reported memory problems from T1 to T4 on one out of the two measures of self‐reported memory problems. For the psychological variables, both the AT group and the CCT group showed extended long‐term effects in burnout compared to MMR, with additional extended long‐term effects on anxiety and work ability in the AT group. The effects on psychological variables were not seen at the 1‐year follow‐up and appeared partly driven by a deterioration in the control group, in conjunction with improvements in the AT group between the 1‐year and 4.5‐year follow‐up. The longitudinal pattern of findings provides stronger support for sustained cognitive effects of CCT than for effects on psychological health, which were less consistent over time for both CCT and AT and should therefore be interpreted more cautiously.

The long‐term effect of CCT on the criterion updating task demonstrated in the 1‐year follow‐up (Gavelin et al. 2018) were maintained at the 4.5‐year follow‐up, with a medium effect size (Cohen's d: 0.77) and no significant change in performance between the 1‐year and the 4.5‐year follow‐ups, indicating a stable long‐term effect. This finding aligns with previous research on cognitive training in healthy populations, where long‐term maintenance on the trained task has been observed, for example at 8 months (Borella et al. 2010), 1.5 years (Sandberg 2014) and even up to 10 years for certain tasks (Rebok et al. 2014). In contrast, studies investigating long‐term effects of cognitive training in clinical populations are scarce. To our knowledge, no prior study has examined outcomes in clinical populations over as long a period as in the present study. However, a shorter‐term follow‐up in a clinical sample with participants diagnosed with depression have shown that CCT effects can persist over time in a cognitive test similar to the trained task (Hoorelbeke et al. 2021). Hence, our results add to previous findings in that the effect of CCT on the criterion task can be sustained in a clinical group up to 4.5 years after the intervention.

Furthermore, our results demonstrated an extended long‐term transfer effect of CCT on both global cognition and episodic memory, which is of clinical importance, because the presence of transfer effects indicate that improvements generalise beyond the trained tasks. Notably, the effect on global cognition remained significant even when episodic memory was excluded from the composite score, indicating that the observed improvement in global cognition was not solely driven by gains in episodic memory. Long‐term transfer effects have been rarely explored in previous research, and when studied, immediate transfer effects often fail to persist over time (Gobet and Sala 2023; Melby‐Lervåg et al. 2016; Rodas et al. 2024). However, there are notable exceptions. Schmiedek et al. (2014) reported lasting improvements in reasoning and episodic memory 2 years after a 100‐day cognitive training programme in young adults. Similarly, Wilkinson and Yang (2020) observed near–near transfer effects 3.5 years following multitask inhibition training in older adults. These findings, together with our own, suggest that under specific conditions, long‐term transfer is not only possible but potentially meaningful in promoting cognitive resilience. While Schmiedek et al. (2014) pointed to the programs breadth, intensity, and duration as potential contributors for the sustained transfer in their study, and Wilkinson and Yang (2020) highlighted factors such as task familiarity and motivation, the mechanisms behind long‐term transfer effects remain unclear. The same uncertainty applies to our findings. Although some have suggested that including strategy training might facilitate transfer (Cicerone et al. 2019; Sigmundsdottir et al. 2016), further research is needed to understand what processes might account for the maintenance of transfer effects over time.

This follow‐up study found no extended long‐term effects of AT on cognitive function. That is, the improvement in episodic memory observed immediately after the intervention (Eskilsson et al. 2017) was not maintained over time, which aligns with the results observed at the 1‐year follow‐up (Gavelin et al. 2018). While previous research has shown that AT positively influence cognitive function (Erickson et al. 2019; Ferrer‐Uris et al. 2022; Stothart et al. 2014), it appears that consistent physical activity over several weeks or months is required for long‐lasting improvements in brain function (Voss et al. 2019). Furthermore, sustained positive effects on episodic memory seem to depend on the continued maintenance of cardiovascular fitness (Hötting et al. 2012). A likely explanation for the absence of long‐term effects in our study is that the participants did not maintain AT over time. Supporting this interpretation, only two out of 16 participants in the AT group reported engaging in more than 2 hours of physical exercise per week at the 4.5‐year follow‐up (data not shown). Considering these findings and previous research, it appears important to design interventions that support long‐term adherence to AT to achieve cognitive benefits. For patients with ED, sustainable AT is supported by flexibility in dosage and frequency, tailored to individual needs (Andersdotter Sandström et al. 2023).

Our results showed that both the CCT and AT groups reported fewer memory problems in everyday life measured with 6‐QEMP. The AT group also showed a tendency for reduced memory problems measured with PRMQ, which did not persist in the sensitivity analyses and should therefore be interpreted with caution. The inconsistency across measures may suggest that improvements are more readily captured by measures that assess memory problems in a stress‐related, context‐sensitive manner, rather than by general questionnaires, highlighting the need for more precise, condition‐specific assessment strategies. Interestingly, both intervention groups showed improvements in everyday cognitive functioning as reflected by self‐reports, whereas improvements in cognitive test performance were observed only in the CCT group. Given the association between psychological health and self‐rated cognition in ED (Nelson et al. 2021), improvements in self‐reported memory in the AT group may parallel the observed effects on self‐rated psychological outcomes, while improvements in the CCT group may be more closely aligned with cognitive training effects. Together, these findings highlight the need to clarify how self‐reported and test‐based cognition relate over time in ED, and whether subjective complaints precede or follow objective recovery.

Moreover, our findings suggest a positive effect of CCT on burnout, consistent with earlier post‐intervention results (Gavelin et al. 2015), but not with 1‐year follow‐up outcomes (Gavelin et al. 2018). The stable burnout levels in the CCT group from T3 to T4, alongside deterioration in the control group, indicate that the effect likely reflects worsening in the control group rather than a lasting benefit of the intervention. We did not find an extended long‐term effect of CCT on anxiety, depression, fatigue or work ability in this study, consistent with the findings from the 1‐year follow‐up (Gavelin et al. 2018). The evidence regarding the effect of CCT on psychological health is mixed and the underlying mechanisms have not been fully understood (Koster et al. 2017). However, one meta‐analysis have shown an effect of CCT on both clinical symptoms and daily life functioning (Motter et al. 2016), and it has been speculated that the mediating effect of CCT on depressive mood is rumination (Koster et al. 2017). Rumination, however, is not considered a core‐symptom of ED (Lindsäter et al. 2023), which may help explain the absence of an effect in our study. On the other hand, ED and depression represent different diagnostic entities, why different mechanisms may be involved in ED. Further research is needed to understand the effects of CCT on psychological health and work ability.

The extended long‐term effects observed for AT on burnout, anxiety, and work ability were unexpected, as they were not present post‐intervention (Eskilsson et al. 2017) or at 1‐year follow‐up (Gavelin et al. 2018). For burnout, the effect appears partly driven by control group deterioration; however, the AT group improved in burnout, anxiety and work ability between T3 and T4. Given the lack of earlier effects and the reported decline in long‐term physical activity, it is unlikely these outcomes stem from the intervention itself. A possible explanation is a selection effect, as dropouts in the AT group may have left a subgroup with higher motivation or resilience not captured by our measures. Importantly, most previous research demonstrates beneficial effects of physical activity for psychological health across populations, with consistent effects on depression, anxiety, and psychological distress (Singh et al. 2023; Wanjau et al. 2023; White et al. 2024). Thus, although the delayed effects of AT on psychological outcomes in this study cannot be confidently attributed to the intervention after 4.5 years, it remains plausible that physical activity contributes to improved psychological health in patients with ED.

The extended long‐term effects of CCT observed in this study on both the trained criterion task as well as transfer to global cognition and episodic memory are encouraging. While these findings must be interpreted with caution due to high attrition and the small sample size (see discussion in *Strengths and Limitations*), they suggest that the cognitive problems associated with ED long‐term may be effectively addressed through targeted intervention such as CCT. This is particularly important given that cognitive problems are reported long‐term in ED (Ellbin et al. 2021; Ellbin, Jonsdottir, Eckerström, et al. 2021) and that cognitive function plays a key role in work ability and everyday life (Gottfredson and Deary 2004; Strenze 2007).

### 4.1 Strengths and Limitations

This study has several notable strengths. First, the extended long‐term follow‐up interval in a clinical sample is unique to our knowledge and adds on to previous knowledge in that effects of CCT can be maintained over 4.5 years in a clinical sample. Secondly, the study was conducted in a clinical setting with participants diagnosed with ED by a physician, enhancing the ecological validity. Third, the comprehensive cognitive test battery used at all assessment times allows for a deeper understanding of the long‐term effects on performance‐based cognitive functioning. The self‐reported measures of cognitive function broadens the understanding of cognitive function over time for ED.

Some limitations should be acknowledged. The relatively small sample size and high attrition may limit generalisability and increase the risk of attrition bias and Type II error. To maintain sensitivity, no correction for multiple comparisons was applied in the primary analyses; however, supplementary BH‐corrected analyses showed that several effects did not survive correction for multiple comparisons. While this may reflect limited power in a modest sample, it nevertheless indicates that the primary analyses should be interpreted with caution.

Attrition is a further limitation. Sensitivity analyses (PMM) indicated potential non‐random dropout in the CCT group for psychological outcomes, while cognitive outcomes and self‐rated cognitive function appeared more robust, with no differential trajectories between completers and non‐completers. This pattern may reflect differences in measurement characteristics, with psychological outcomes being more state‐dependent and cognitive measures capturing more stable aspects of functioning; however, this interpretation should be made with caution. Furthermore, although the results of CCT on cognitive function were stable, the differences in psychological health trajectories suggest that a subgroup with more favourable long‐term psychological outcomes remained in the CCT group, potentially limiting generalisability of the findings. However, dropout analyses indicated no baseline differences at T1, apart from a small difference in verbal ability. Differences instead emerged at later time points. Whether these differences reflect intervention effects or are partly driven by selective dropout remains uncertain. Overall, these findings underscore the importance of interpreting the results with caution, particularly for outcomes related to psychological health.

### 4.2 Research and Clinical Implications

The current findings offer both research and clinical insights on how to support cognition in exhaustion disorder (ED), where CCT shows extended long‐term benefits not evident following AT. While the results are promising, the mechanisms underlying the positive effects of CCT remain insufficiently understood. Future research should focus on clarifying these mechanisms, particularly by accounting for individual differences. Single‐case designs, which allow for the exploration of individual trajectories during training, may be valuable in this context. Cognitive function is a prevalent issue for patients with ED. The present findings highlight the potential value of addressing cognitive functioning as part of treatment of ED, to reduce persistent cognitive problems. This is likely also relevant for other stress‐related conditions, given the substantial overlap between ED and related disorders. Notably, AT demonstrated beneficial effects immediately post‐intervention, but these were not sustained at follow‐up, coinciding with reduced physical activity within the group. Considering the broader literature linking physical activity to improved cognitive function, it is possible that continued engagement in physical activity is necessary to maintain benefits over time. Accordingly, future research and clinical care should focus on how to best support long‐term adherence to physical activity in this population. The high dropout rates and the pattern in dropouts suggest that timing is important when offering demanding interventions such as CCT and AT, while also calling for caution when interpreting the findings.

## 5 Conclusion

Our findings suggest that improvements in cognitive function following cognitive training can be maintained over time in patients with ED. Although an improvement of AT was observed for psychological health and work ability, these effects emerged between the 1‐ and 4.5‐year follow‐ups and should be interpreted cautiously given the high attrition.

## Author Contributions

**Ingela Aronsson:** conceptualisation, methodology, investigation, formal analysis, visualisation, writing – original draft, and writing – review and editing. **Anna Stigsdotter Neely:** conceptualisation, methodology, funding acquisition, investigation, formal analysis, supervision, and writing – review and editing. **Carl‐Johan Boraxbekk:** conceptualisation, methodology, and writing – review and editing. **Therese Eskilsson:** conceptualisation, methodology, and writing – review and editing. **Hanna M. Gavelin:** conceptualisation, methodology, funding acquisition, investigation, formal analysis, supervision, and writing – review and editing.

## Funding

This research was supported by the Swedish Research Council for Health, Working life and Welfare (Grant No. 2009‐0772) awarded to Anna Stigsdotter Neely and (Grant No. 2010‐01111) awarded to Hanna M. Gavelin and by AFA Försäkring (Grant No. 150175) awarded to Anna Stigsdotter Neely and by the Swedish Social Insurance Agency (REHSAM, Grant No. 99368‐2009/RS09) and the Västerbotten County Council awarded to Lisbeth Järvholm.

## Ethics Statement

The study followed the guidelines of the Declaration of Helsinki and was approved by the Umeå Regional Ethical Review Board (Dnr 2015–475‐ 32 M addition to 2010–53‐31 M). Study design: The study was retrospectively registered at ClinicalTrials.gov in 2017 (Identifier: NCT0073772). The study plan is available on the registry.

## Conflicts of Interest

The authors declare no conflicts of interest.

## Supporting information


Supporting Information S1

Supporting Information S2

Supporting Information S3

Supporting Information S4

Supporting Information S5

Supporting Information S6

## Data Availability

The data that support the findings of this study are available on request from the corresponding author. The data are not publicly available due to privacy or ethical restrictions.

## Appendix 1

### Diagnostic Criteria for Exhaustion Disorder According to the Swedish National Board of Health and Welfare

**Table:**

A. Physical and mental symptoms of exhaustion with minimum of 2 weeks duration. The symptoms have developed in response to one or more identifiable stressors which have been present for at least 6 months. B. Markedly reduced mental energy, which is manifested by reduced initiative, lack of endurance or increase of time needed for recovery after mental efforts. C. At least four of the following symptoms have been present most of the day, nearly every day, during the same 2‐week period: 1. Persistent complaints of impaired memory or concentration 2. Markedly reduced capacity to tolerate demands or to work under time pressure 3. Emotionally instability or irritability 4. Disturbed sleep 5. Persistent complaints of physical weakness or fatigue 6. Physical symptoms such as muscular pain, chest pain, palpitations, gastrointestinal problems, vertigo or increased sensitivity to sounds D. The symptoms cause clinically significant distress or impairment in occupational, social or other important areas of functioning. E. The symptoms are not due to the direct physiological effects of a substance (e.g. drug abuse, medication) or a general medical condition (e.g. hypothyroidism, diabetes, infectious disease). F. If criteria for major depressive disorder, dysthymic disorder or generalised anxiety disorder are met, ED is set as co‐morbid condition.

*Note:* All capital letters must be fulfiled for the diagnosis.

## Appendix 2

### Sample Characteristics at Baseline

**Table:**

Variable	Control group (*n* = 41)	Cognitive training (*n* = 44)	Aerobic training (*n* = 47)
Sex,			
female/male, *n*	38/3	34/10	39/8
Age			
Mean (SD)	41.88 (7.41)	43.89 (9.21)	44.15 (8.60)
Range	22–58	25–58	25–60
Education, *n* (%)			
University	26 (63%)	25 (57%)	29 (62%)
Sick leave before MMR, *n* (%)			
No sick leave	10 (24%)	4 (9%)	6 (13%)
≤ 6 month	20 (49%)	25 (57%)	30 (64%)
7–12 months	5 (12%)	6 (14%)	6 (13%)
12–24 months	3 (7%)	3 (7%)	2 (4%)
> 24 months	3 (7%)	6 (13%)	3 (6%)
Problems with memory and concentration^a^			
None/negligible	0 (0%)	0 (0%)	0 (0%)
Small	1 (2.5%)	1 (3%)	1 (2%)
Moderate	10 (25%)	15 (43%)	9 (20%)
Severe/pronounced	29 (72.5%)	19 (54%)	36 (78%)
Physical exercise, *n* (%)			
≤ 2 hours/week	34 (85%)	29 (83%)	37 (80%)
> 2 hours/week	6 (15%)	6 (17%)	9 (20%)
Verbal ability, mean (SD)^b^	22.83 (4.78)	21.46 (4.73)	22.87 (3.97)

*Note:* Number of respondents varies for different variables; treatment as usual: *n =* 40–41; cognitive training group: *n* = 35–44; aerobic training group: *n* = 45–47.

^a^ Based on the question “For the past 3 months, have you experienced any of the following problems: (1) Problems with sleep; (2) Problems with memory or concentration. If so, rate how severe these problems have been: negligible; small; moderate; severe; pronounced.”

^b^ SRB:1.

## Appendix 3

### The Computerised Cognitive Training Programme

The cognitive training battery consisted of six different tasks, addressing the cognitive functions updating, shifting, visuospatial short‐term memory and episodic memory. The shifting tasks were alternated at each training session, hence, five tasks were conducted at each session. All tasks, except for the shifting tasks, were adaptive. For a full description of the training battery, see Gavelin et al. (2015).

**Table:**

Cognitive function	Task	Description of task
Updating	Letter memory running span	A list of letters was presented at a pace of 2 s/letter. The participants were asked to recall the four last letters seen.
	Keep track task	A list of words was presented for the participants at a pace of 2 s/word, as well as 3−5 semantic categories. The task for the participants was to remember the last word within each category.
Shifting	Alternating run with digits	Digits were presented in a square and the task for the participants was to classify the digits as odd/even or high/low depending on where in the square they were presented.
	Alternating run with letters	Letters were presented in combination with a blue square or red circle. The task for the participants was to classify the letters as beginning/end of alphabet or vowel/consonant, depending on the accompanied figure.
Visuospatial short‐term memory	Visuospatial span task	A grid with 4 × 4 squares served as the base, and sequences of flashing squares were presented for the participant. The task was to remember the order the flashing squares were presented.
Episodic memory	Three‐word‐association task	12 triplets of words were presented: a location, an object and a colour. Following the presentation of all three‐word combinations, the task was to recall the object and colour using the location as retrieval cue.
