# Supplementary material for: Extended Long‐Term Effects of Cognitive and Aerobic Training on Cognitive Function in Patients With Stress‐Related Exhaustion Disorder: A 4.5‐Year Follow‐Up of a Randomized Controlled Trial
Source: Stress Health. 2026 Sep 4;42(5):e70218. doi: 10.1002/smi.70218 (PMC13545551; doi:10.1002/smi.70218)
Supplement: Supplementary file 1 [file SMI-42-e70218-s001.docx]

**Supplementary file E**

*Results From the Mixed Models on Psychological Variables, Self-rated Cognition, and Work Ability*

| **Outcome** | **SMBQ**  *β (SE)* | **HADS depression**  *β (SE)* | **HADS**  **anxiety**  *β (SE)* | **CIS**  *β (SE)* | **PRMQ**  *β (SE)* | **6-QEMP**  *β (SE)* | **WAI**  *β (SE)* |
| --- | --- | --- | --- | --- | --- | --- | --- |
| **Fixed effects** |  |  |  |  |  |  |  |
| Intercept | 4.78 (0.18)*** | 7.40 (0.56)*** | 9.56 (0.62)*** | 96.20 (3.54)*** | 49.40 (1.80) | 20.50 (0.62) | 27.30 (1.12)*** |
| *Time (ref. T1)* |  |  |  |  |  |  |  |
| T2 | -0.44 (0.17)** | -1.35 (0.53)* | -1.16 (0.60) | -9.26 (3.45) | -0.92 (2.05) | 0.38 (0.82) | 2.88 (1.16)* |
| T3 | -0.85 (0.17)*** | -2.15 (0.53)*** | -2.38 (0.60)*** | -16.50 (3.48)*** |  |  | 5.94 (1.22)*** |
| T4 | -0.43 (0.21)* | -2.16 (0.66)*** | -1.08 (0.75) | -15.50 (4.31)*** | -1.96 (1.93) | 0.05 (0.76) | 5.38 (1.40)*** |
| *Group (ref. control)* |  |  |  |  |  |  |  |
| Cognitive training | 0.22 (0.25) | -0.70 (0.78) | 0.80 (0.87) | -0.30 (4.97) | 0.43 (2.50) | 1.05 (0.87) | -0.99 (1.58) |
| Aerobic training | 0.04 (0.24) | -0.79 (0.76) | -0.42 (0.85) | -3.40 (4.84) | 1.31 (2.54) | 0.95 (0.87) | -0.58 (1.53) |
| *Group x Time* |  |  |  |  |  |  |  |
| Cognitive training x T2 | -0.36 (0.23) | -0.62 (0.75) | -1.45 (0.85) | -1.75 (4.91) | -4.26 (2.70) | -3.33 (1.06) | 0.66 (1.69) |
| Cognitive training x T3 | -0.20 (0.24) | -0.01 (0.76) | -0.30 (0.86) | 0.57 (4.94) | — | — | 2.09 (1.80) |
| Cognitive training x T4 | -0.69 (0.30)* | -0.50 (0.95) | -1.61 (1.08) | -7.76 (6.23) | -3.07 (2.78) | -2.77 (1.09) | 2.94 (1.99) |
| Aerobic training x T2 | -0.33 (0.23) | -0.26 (0.73) | -1.06 (0.83) | -3.94 (4.79) | - 4.12 (3.00) | -2.04 (1.22) | 1.88 (1.74) |
| Aerobic training x T3 | 0.06 (0.23) | 0.44 (0.73) | -0.93 (0.83) | 5.91 (4.81) | — | — | -1.23 (1.87) |
| Aerobic training x T4 | -1.04 (0.31)*** | -1.17 (0.98) | -2.59 (1.11)* | -8.84 (6.41) | -6.47 (2.94) | -4.20 (1.15) | 6.74 (2.09)** |
| **Random effects^a, b^** |  |  |  |  |  |  |  |
| Intercept variance | 0.69 (0.83) | 6.62 (2.57) | 7.89 (2.81) | 250.20 (15.82) | 78.54 (8.86) | 7.59 (2.76) | 25.20 (5.02) |
| Residual variance | 0.54 (0.73) | 5.47 (2.34) | 7.01 (2.65) | 234.60 (15.32) | 40.96 (6.40) | 6.58 (2.56) | 22.52 (4.75) |

*Note*: * p< 0.05; ** p < 0.01; *** p < 0.001. SMBQ = Shirom-Melamed Burnout Questionnaire. HADS = Hospital Anxiety and Depression Scale. CIS = Checklist Individual Strength. WAI = Work Ability Index.^a^ No p-values calculated, ^b^. Values are presented as mean (SD) instead of standard error (SE).
