# Supplementary material for: Extended Long‐Term Effects of Cognitive and Aerobic Training on Cognitive Function in Patients With Stress‐Related Exhaustion Disorder: A 4.5‐Year Follow‐Up of a Randomized Controlled Trial
Source: Stress Health. 2026 Sep 4;42(5):e70218. doi: 10.1002/smi.70218 (PMC13545551; doi:10.1002/smi.70218)
Supplement: Supplementary file 2 [file SMI-42-e70218-s002.docx]

**Supplementary file A**

**Supplementary Table A1**

*Available data for each cognitive test across assessment time, %*

**Supplementary Table A2**

*Available data for each questionnaire across assessment time, %*
