# Supplementary material for: Extended Long‐Term Effects of Cognitive and Aerobic Training on Cognitive Function in Patients With Stress‐Related Exhaustion Disorder: A 4.5‐Year Follow‐Up of a Randomized Controlled Trial
Source: Stress Health. 2026 Sep 4;42(5):e70218. doi: 10.1002/smi.70218 (PMC13545551; doi:10.1002/smi.70218)
Supplement: Supplementary file 3 [file SMI-42-e70218-s003.docx]

**Supplementary File D**

*Results From the Mixed Models on the Cognitive Variables*

|  | **Global cognition**  *β (SE)* | **Executive function**  *β (SE)* | **Working memory**  *β (SE)* | **Episodic memory**  *β (SE)* | **Perceptual speed**  *β (SE)* | **Reasoning ability**  *β (SE)* | **Trained updating task**  *β (SE)* |
| --- | --- | --- | --- | --- | --- | --- | --- |
| **Fixed effects** |  |  |  |  |  |  |  |
| Intercept | 0.12 (0.10) | 0.14 (0.11) | 0.07 (0.13) | 0.15 (0.17) | 0.23 (0.17) | -0.04 (0.17) | 0.08 (0.17) |
| *Time (ref. T1)* |  |  |  |  |  |  |  |
| T2 | 0.10 (0.06) | 0.33 (0.09)*** | 0.01 (0.10) | -0.03 (0.14) | 0.41 (0.12)*** | -0.35 (0.16)* | 0.14 (0.20) |
| T3 | 0.25 (0.06)*** | 0.37 (0.10)*** | 0.12 (0.11) | 0.22 (0.15) | 0.54 (0.13)*** | 0.18 (0.17) | 0.22 (0.21) |
| T4 | 0.19 (0.07)** | 0.43 (0.11)*** | 0.13 (0.12) | 0.18 (0.17) | 0.20 (0.14) | -0.13 (0.19) | 0.18 (0.23) |
| *Group (ref. control)* |  |  |  |  |  |  |  |
| Cognitive training | -0.19 (0.14) | -0.24 (0.16) | -0.03 (0.19) | -0.24 (0.24) | -0.38 (0.23) | -0.11 (0.23) | -0.20 (0.23) |
| Aerobic training | -0.20 (0.14) | -0.21 (0.15) | -0.18 (0.18) | -0.24 (0.23) | -0.30 (0.23) | 0.20 (0.23) | -0.02 (0.23) |
| *Group x Time* |  |  |  |  |  |  |  |
| Cognitive training x T2 | 0.27 (0.08)** | 0.11 (0.14) | 0.23 (0.15) | 0.46 (0.21)* | 0.13 (0.17) | 0.66 (0.24)** | 1.19 (0.28)*** |
| Cognitive training x T3 | 0.21 (0.09)* | 0.04 (0.15) | 0.25 (0.16) | 0.30 (0.23) | 0.22 (0.19) | 0.28 (0.26) | 0.87 (0.30)** |
| Cognitive training x T4 | 0.25 (0.10)* | 0.12 (0.16) | 0.16 (0.17) | 0.68 (0.24)** | 0.26 (0.20) | 0.15 (0.27) | 0.77 (0.33)* |
| Aerobic training x T2 | 0.16 (0.09) | -0.00 (0.14) | 0.15 (0.15) | 0.59 (0.22)** | 0.14 (0.18) | 0.08 (0.24) | -0.43 (0.29) |
| Aerobic training x T3 | 0.07 (0.09) | 0.10 (0.15) | -0.03 (0.16) | 0.33 (0.23) | 0.12 (0.19) | -0.12 (0.26) | 0.10 (0.31) |
| Aerobic training x T4 | 0.08 (0.10) | -0.02 (0.16) | -0.01 (0.18) | 0.39 (0.25) | 0.25 (0.21) | -0.06 (0.28) | 0.25 (0.34) |
| **Random effects** **^a, b^** |  |  |  |  |  |  |  |
| Intercept variance | 0.31 (0.56) | 0.34 (0.58) | 0.53 (0.73) | 0.78 (0.89) | 0.88 (0.94) | 0.63 (0.79) | 0.44 (0.66) |
| Residual variance | 0.05 (0.22) | 0.14 (0.37) | 0.16 (0.41) | 0.33 (0.57) | 0.23 (0.48) | 0.43 (0.66) | 0.65 (0.81) |

*Note*: * p< 0.05; ** p < 0.01; *** p < 0.001, ^a^ No p-values calculated, ^b^. Values are presented as mean (SD) instead of standard error (SE).
