# Supplementary material for: Extended Long‐Term Effects of Cognitive and Aerobic Training on Cognitive Function in Patients With Stress‐Related Exhaustion Disorder: A 4.5‐Year Follow‐Up of a Randomized Controlled Trial
Source: Stress Health. 2026 Sep 4;42(5):e70218. doi: 10.1002/smi.70218 (PMC13545551; doi:10.1002/smi.70218)
Supplement: Supplementary file 4 [file SMI-42-e70218-s004.docx]

| **Variable** | **Group** | **T1 to T4** |  |  |  |
| --- | --- | --- | --- | --- | --- |
|  | (ref. control group) |  |  |  |  |
|  |  | *β (95% CI)* | *p-value* | *p-value B.H* | *Sig. B.H.* |
| **Global cognitive score** | Cognitive training | 0.25 (0.06, 0.44) | **.011** | .074 | No |
|  | Aerobic training | 0.08 (-0.12, 0.27) | .438 | .643 | No |
| **Executive function** | Cognitive training | 0.12 (-0.20, 0.43) | .459 | .643 | No |
|  | Aerobic training | -0.02 (-0.34, 0.30) | .894 | .935 | No |
| **Working memory** | Cognitive training | 0.16 (-0.18, 0.49) | .358 | .643 | No |
|  | Aerobic training | -0.01 (-0.36, 0.33) | .935 | .935 | No |
| **Episodic memory** | Cognitive training | 0.68 (0.21, 1.15) | **.005** | .071 | No |
|  | Aerobic training | 0.39 (-0.10, 0.88) | .116 | .404 | No |
| **Perceptual speed** | Cognitive training | 0.26 (-0.14, 0.66) | .195 | .546 | No |
|  | Aerobic training | 0.25 (-0.17, 0.66) | .247 | .576 | No |
| **Reasoning ability** | Cognitive training | 0.15 (-0.39, 0.69) | .582 | .741 | No |
|  | Aerobic training | -0.06 (-0.62, 0.49) | .821 | .935 | No |
| **Cognitive training criterion task** | Cognitive training | 0.77 (0.13, 1.42) | **.019** | .089 | No |
|  | Aerobic training | 0.25 (-0.42, 0.92) | .457 | .643 | No |

**Supplementary file F**

**Supplementary Table F1**

*Results From the Benjamini-Hochberg Correction on the Cognitive Variables*

**Supplementary Table F2**

*Results From the Benjamini-Hochberg Correction on the Psychological Variables*

| **Variable** | **Group**  (ref. control group) | **T1 to T4** | | |  |
| --- | --- | --- | --- | --- | --- |
|  |  | *Estimate (95% CI)* | *p-value* | *p-value B.H* | *Sig. B.H.* |
| **SMBQ** | Cognitive training | -0.69 (-1.27, -0.10) | **.022** | .052 | No |
|  | Aerobic training | -1.04 (-1.65, -0.44) | **<.001** | **.005** | Yes |
| **HADS** |  |  |  |  |  |
| Depression | Cognitive training | -0.50 (-2.35, 1.34) | .592 | .596 | No |
|  | Aerobic training | -1.17 (-3.07, 0.73) | .228 | .273 | No |
| Anxiety | Cognitive training | -1.61 (-3.70, 0.48) | .130 | .221 | No |
|  | Aerobic training | -2.59 (-4.74, -0.44) | **.018** | .052 | No |
| **CIS** | Cognitive training | -7.76 (-20.00, 4.50) | .214 | .272 | No |
|  | Aerobic training | -8.84 (-21.50, 3.78) | .169 | .237 | No |
| **PRMQ^a^** | Cognitive training | -3.07 (-8.58, 2.44) | .272 | .293 | No |
|  | Aerobic training | -6.47 (-12.30, -0.64) | **.030** | .060 | No |
| **6-QEMP^a^** | Cognitive training | -2.77 (-4.92, -0.61) | **.012** | **.044** | Yes |
|  | Aerobic training | -4.20 (-6.47, -1.93) | **<.001** | **.005** | Yes |
| **WAI** | Cognitive training | 2.94 (-0.99, 6.86) | .142 | .221 | No |
|  | Aerobic training | 6.74 (2.62, 10.90) | **.001** | **.007** | Yes |
