# Supplementary material for: Extended Long‐Term Effects of Cognitive and Aerobic Training on Cognitive Function in Patients With Stress‐Related Exhaustion Disorder: A 4.5‐Year Follow‐Up of a Randomized Controlled Trial
Source: Stress Health. 2026 Sep 4;42(5):e70218. doi: 10.1002/smi.70218 (PMC13545551; doi:10.1002/smi.70218)
Supplement: Supplementary file 5 [file SMI-42-e70218-s005.docx]

**Supplementary File B**

*Example of R-code*

#Activated packages

library(dplyr)	# For data manipulation

library(lme4)	# For fitting linear mixed-effects models (lmer)

library(lmerTest)	# For p-values and degrees of freedom in lmer

library(emmeans)	# For estimated marginal means and contrast tests

library(broom.mixed)	#

library(knitr)	#

library(ggplot2)	# For visualization of the results

\# Mixed models

model_global_cognition <- lmer(

global_cognition ~ Assessment_time * Randkod + (1|IDNR),

data = data

)

summary(model_global_cognition)

confint(model_global_cognition, method = "Wald")

results_gc <- tidy(model_global_cognition, effects = "fixed", conf.int = TRUE, conf.level = 0.95)

results_gc

\# To calculate estimated marginal means

emm_gc <- emmeans(model_global_cognition, ~ Randkod * Assessment_time)

contrast_results_gc <- contrast(

emm_gc,

method = list("T4 - T3" = c(0, 0, -1, 1)),

by = "Randkod",

infer = c(TRUE, TRUE)

)

summary(contrast_results_gc)
