# Supplementary material for: Extended Long‐Term Effects of Cognitive and Aerobic Training on Cognitive Function in Patients With Stress‐Related Exhaustion Disorder: A 4.5‐Year Follow‐Up of a Randomized Controlled Trial
Source: Stress Health. 2026 Sep 4;42(5):e70218. doi: 10.1002/smi.70218 (PMC13545551; doi:10.1002/smi.70218)
Supplement: Supplementary file 6 [file SMI-42-e70218-s006.docx]

**Supplementary file C.**

*Dropout Analyses for the Entire Sample*

| Variable | Entire sample | | | |
| --- | --- | --- | --- | --- |
|  | Completers | Non-completers | t/χ² | p-value |
| Age | 42.13 (8.67) | 44.26 (8.24) | 1.43 | .078 |
|  | *n* = 56 | *n* = 76 |  |  |
| Sex (f/m) | 48/8 | 63/13 | χ²=0.192 | .662 |
| Educational level |  |  |  |  |
| Compulsory school | 1 | 5 |  |  |
| Secondary school | 18 | 28 |  |  |
| University/higher edu | 37 | 43 |  |  |
|  |  |  | χ²=2.31 | .315 |
| Verbal ability | 23.34 (3.07) | 21.65 (5.27) | -2.26 | .013 |
|  | *n* = 56 | *n* = 71 |  |  |

**Supplementary file C continued.**

*Dropout Analyses by Intervention Group*

| Variable | MMR | | | |  | CCT | | | |  | AT | | |  |
| --- | --- | --- | --- | --- | --- | --- | --- | --- | --- | --- | --- | --- | --- | --- |
|  | Completers | Non-completers | t/χ² | p-value |  | Completers | Non-completers | t/χ² | p-value |  | Completers | Non-completers | t/χ² | p-value |
| Age | 40.33 (7.91) | 43.94 (6.61) | 1.53 | 0.134 |  | 43.58 (9.20) | 45.00 (9.76) | 0.462 | 0.647 |  | 44.06 (8.84) | 45.37 (8.69) | 0.472 | 0.640 |
|  | *n* = 21 | *n* = 17 |  |  |  | *n* = 19 | *n* = 19 |  |  |  | *n* = 16 | *n* = 27 |  |  |
| Sex (f/m) | 20/1 | 1/0 | χ²=0.83 | 0.362 |  | 12/7 | 17/2 | χ²=3.64 | 0.056 |  | 15/1 | 20/7 | χ²=2.57 | 0.109 |
| Educational level |  |  |  |  |  |  |  |  |  |  |  |  |  |  |
| Compulsory school | 0 | 1 |  |  |  | 1 | 2 |  |  |  | 0 | 2 |  |  |
| Secondary school | 7 | 7 |  |  |  | 7 | 9 |  |  |  | 4 | 12 |  |  |
| University/higher edu | 14 | 12 |  |  |  | 11 | 14 |  |  |  | 12 | 17 |  |  |
|  |  |  | χ²=1.13 | 0.568 |  |  |  | χ²=0.13 | 0.938 |  |  |  | χ²=2.31 | 0.315 |
| Verbal ability | 23.90 (3.25) | 21.63 (5.90) | -1.49 | 0.148 |  | 22.21 (3.12) | 20.77 (5.77) | -1.01 | 0.319 |  | 23.94 (2.49) | 22.30 (4.50) | -1.59 | 0.119 |
|  | *n* = 21 | *n* = 19 |  |  |  | *n* = 19 | *n* = 22 |  |  |  | *n* =16 | *n* = 30 |  |  |

| Burnout |  |  |  |  |  |  |  |  |  |  |  |  |  |  |
| --- | --- | --- | --- | --- | --- | --- | --- | --- | --- | --- | --- | --- | --- | --- |
| T1 | 5.00 (0.83) | 4.50 (1.19) | -1.49 | 0.074 |  | 4.94 (0.89) | 5.02 (0.89) | 0.29 | 0.775 |  | 4.87 (1.00) | 4.87 (0.94) | -0.00 | 0.998 |
|  | *n* = 21 | *n* = 17 |  |  |  | *n* = 19 | *n* = 19 |  |  |  | *n* = 16 | *n* = 27 |  |  |
| T2 | 4.64 (1.01) | 4.01 (1.07) | -1.92 | 0.062 |  | 3.75 (0.84) | 4.55 (1.09) | 2.74 | 0.009 |  | 3.94 (1.01) | 4.11 (1.33) | 0.50 | 0.619 |
|  | *n* = 21 | *n* = 19 |  |  |  | *n* = 19 | *n* = 25 |  |  |  | *n* = 16 | *n* = 30 |  |  |
| T3 | 4.26 (1.18) | 3.58 (1.25) | -1.76 | 0.087 |  | 3.38 (0.97) | 4.37 (0.86) | 3.33 | 0.002 |  | 3.94 (1.36) | 4.11 (1.25) | 0.40 | 0.695 |
|  | *n* = 21 | *n* = 19 |  |  |  | *n* = 17 | *n* = 23 |  |  |  | *n* = 16 | *n* = 29 |  |  |
| Depression |  |  |  |  |  |  |  |  |  |  |  |  |  |  |
| T1 | 7.47 (3.50) | 7.00 (3.54) | -0.41 | 0.682 |  | 6.68 (2.54) | 6.84 (2.91) | 0.18 | 0.860 |  | 6.94 (4.48) | 6.74 (3.41) | -0.15 | 0.881 |
|  | *n* = 21 | *n* = 17 |  |  |  | *n* = 19 | *n* = 19 |  |  |  | *n* = 16 | *n* = 27 |  |  |
| T2 | 6.57 (4.49) | 5.47 (3.76) | -0.84 | 0.406 |  | 4.32 (2.33) | 5.04 (3.16) | 0.88 | 0.387 |  | 4.19 (3.25) | 5.33 (4.12) | 1.04 | 0.307 |
|  | *n* = 21 | *n* = 19 |  |  |  | *n* = 19 | *n* = 25 |  |  |  | *n* = 16 | *n* = 30 |  |  |
| T3 | 5.76 (4.65) | 4.68 (3.48) | -0.84 | 0.409 |  | 3.47 (2.07) | 5.39 (2.82) | 2.48 | 0.017 |  | 4.81 (3.82) | 4.83 (3.46) | 0.01 | 0.990 |
|  | *n* = 21 | *n* = 19 |  |  |  | *n* = 17 | *n* = 23 |  |  |  | *n* = 16 | *n* = 29 |  |  |
| Anxiety |  |  |  |  |  |  |  |  |  |  |  |  |  |  |
| T1 | 9.89 (3.70) | 8.82 (3.99) | -0.85 | 0.404 |  | 10.47 (2.70) | 10.47 (3.41) | 0.00 | 1.000 |  | 9.19 (4.18) | 9.19 (3.69) | 0.01 | 0.995 |
|  | *n* = 21 | *n* = 17 |  |  |  | *n* = 19 | *n* = 19 |  |  |  | *n* = 16 | *n* = 27 |  |  |
| T2 | 8.52 (3.89) | 8.26 (3.51) | -0.22 | 0.825 |  | 7.68 (2.89) | 7.80 (3.39) | 0.12 | 0.903 |  | 7.13 (3.05) | 6.73 (3.61) | -0.39 | 0.700 |
|  | *n* = 21 | *n* = 19 |  |  |  | *n* = 19 | *n* = 25 |  |  |  | *n* = 16 | *n* = 30 |  |  |
| T3 | 8.00 (4.88) | 6.26 (3.46) | -1.31 | 0.199 |  | 7.29 (2.95) | 8.00 (4.72) | 0.58 | 0.565 |  | 7.31 (4.84) | 7.86 (4.75) | 0.37 | 0.716 |
|  | *n* = 21 | *n* = 19 |  |  |  | *n* = 17 | *n* = 23 |  |  |  | *n* = 16 | *n* = 29 |  |  |
| Fatigue |  |  |  |  |  |  |  |  |  |  |  |  |  |  |
| T1 | 101.05 (16.54) | 89.82 (21.82) | -1.75 | 0.090 |  | 94.95 (15.91) | 96.71 (20.04) | 0.30 | 0.766 |  | 92.06 (23.79) | 95.53 (16.72) | 0.51 | 0.613 |
|  | *n* = 21 | *n* = 17 |  |  |  | *n* = 19 | *n* = 19 |  |  |  | *n* = 16 | *n* = 27 |  |  |
| T2 | 95.33 (21.19) | 77.63 (20.79) | -2.67 | 0.011 |  | 76.00 (16.73) | 92.14 (21.70) | 2.75 | 0.009 |  | 74.94 (18.44) | 82.00 (23.47) | 1.12 | 0.269 |
|  | *n* = 21 | *n* = 19 |  |  |  | *n* = 19 | *n* = 24 |  |  |  | *n* = 16 | *n* = 30 |  |  |
| T3 | 87.52 (25.19) | 71.05 (20.92) | -2.26 | 0.030 |  | 68.06 (18.51) | 88.61 (17.16) | 3.58 | 0.001 |  | 80.44 (29.59) | 83.03 (25.30) | 0.30 | 0.769 |
|  | *n* = 21 | *n* = 19 |  |  |  | *n* = 17 | *n* = 23 |  |  |  | *n* = 16 | *n* = 29 |  |  |

*Note.* The table presents the detailed results from the drop-out analysis conducted on the whole sample and separately within each intervention group. These analyses include baseline demographic, psychological health, and cognitive performance variables at T1, T2, and T3.
